# Species and gene divergence in *Littorina* snails detected by array comparative genomic hybridization

**DOI:** 10.1186/1471-2164-15-687

**Published:** 2014-08-18

**Authors:** Marina Panova, Tomas Johansson, Björn Canbäck, Johan Bentzer, Magnus Alm Rosenblad, Kerstin Johannesson, Anders Tunlid, Carl André

**Affiliations:** Department of Biological and Environmental Sciences - Tjärnö, Gothenburg University, Gothenburg, Sweden; Department of Biology, Microbial Ecology Group, Lund University, Lund, Sweden; Department of Chemistry and Molecular Biology, Gothenburg University, Gothenburg, Sweden

**Keywords:** Comparative genomic hybridization, Oligonucleotide arrays, *Littorina*, Ecotypes, Genome evolution, Gene divergence

## Abstract

**Background:**

Array comparative genomic hybridization (aCGH) is commonly used to screen different types of genetic variation in humans and model species. Here, we performed aCGH using an oligonucleotide gene-expression array for a non-model species, the intertidal snail *Littorina saxatilis.* First, we tested what types of genetic variation can be detected by this method using direct re-sequencing and comparison to the *Littorina* genome draft. Secondly, we performed a genome-wide comparison of four closely related *Littorina* species: *L. fabalis*, *L. compressa*, *L. arcana* and *L. saxatilis* and of populations of *L. saxatilis* found in Spain, Britain and Sweden. Finally, we tested whether we could identify genetic variation underlying “Crab” and “Wave” ecotypes of *L. saxatilis.*

**Results:**

We could reliably detect copy number variations, deletions and high sequence divergence (i.e. above 3%), but not single nucleotide polymorphisms. The overall hybridization pattern and number of significantly diverged genes were in close agreement with earlier phylogenetic reconstructions based on single genes. The trichotomy of *L. arcana*, *L. compressa* and *L. saxatilis* could not be resolved and we argue that these divergence events have occurred recently and very close in time. We found evidence for high levels of segmental duplication in the *Littorina* genome (10% of the transcripts represented on the array and up to 23% of the analyzed genomic fragments); duplicated genes and regions were mostly the same in all analyzed species. Finally, this method discriminated geographically distant populations of *L. saxatilis*, but we did not detect any significant genome divergence associated with ecotypes of *L. saxatilis.*

**Conclusions:**

The present study provides new information on the sensitivity and the potential use of oligonucleotide arrays for genotyping of non-model organisms. Applying this method to *Littorina* species yields insights into genome evolution following the recent species radiation and supports earlier single-gene based phylogenies. Genetic differentiation of *L. saxatilis* ecotypes was not detected in this study, despite pronounced innate phenotypic differences. The reason may be that these differences are due to single-nucleotide polymorphisms.

**Electronic supplementary material:**

The online version of this article (doi:10.1186/1471-2164-15-687) contains supplementary material, which is available to authorized users.

## Background

Changes in genes and genomes associated with permanent splits of evolutionary lineages contribute key information for our understanding of the evolution of new species [1]. Following speciation, large parts of the genomes still have paraphyletic genealogies but, with time, an increasing number of genes will convert to monophyly by lineage sorting [2]. However, lineage sorting takes time, particularly if populations are large, and the reciprocal monophyly criterion cannot be applied for species delimitation in recent radiations [3, 4]. In addition, it is now widely accepted that conflicting gene genealogies may exist within a given species tree, which complicates phylogenetic inferences and may even lead to an incorrect species tree [5, 6]. In current approaches, phylogenetic and phylogeographic inferences more and more often rely on a large number of genes, sampled across the genome, *e.g.* [7–9]. Nevertheless, the vast majority of phylogenies published recently using molecular systematics, for various groups of organisms, are based on one or very few genes. We can now test and pose a timely question: whether we can trust phylogenies based on mtDNA and single nuclear gene variation in the era of genomics. In particular, the utility of mitochondrial DNA (mtDNA) markers for phylogenetic and phylogeographic inferences has been questioned, *e.g.* [10, 11].

In addition to random variation in lineage sorting across the genome, diversifying selection is another process leading to discordance between genealogies and the true phylogeny of lineages [12–14]. Genes that contribute to barriers against gene flow between incipient or recently separated species will evolve at higher rates compared to neutral genomic regions [15, 16]. Further, these genes may drive divergence of surrounding genomic regions [17–19]. Genome-wide approaches have recently been used to identify loci with elevated degrees of divergence in several systems, *e.g*. [20–23]. Closely related lineages with various splitting times are of special interest in studying the progress of genome evolution [22]. Parallel processes of divergence in demographically independent systems give an opportunity to test whether the same or alternative genomic architectures have been used in repeated adaptations to similar environments [24].

**Figure 1:**
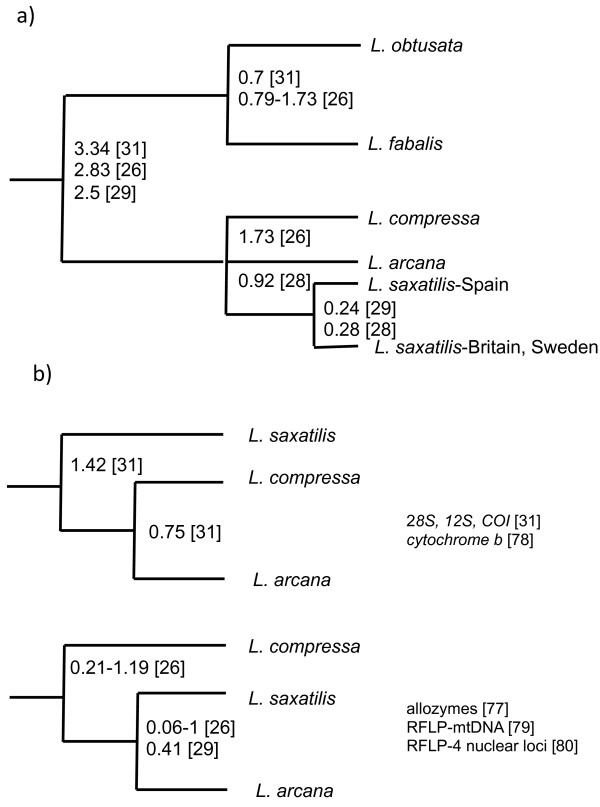
**Phylogeny of five North-Atlantic species of** ***Littorina*** **subgenus** ***Neritrema.*** **a)** Dendrogram representing the phylogeny of the five North-Atlantic species of *Littorina*, subgenus *Neritrema*. Numbers indicate divergence times in Ma, estimated from molecular data in previous studies. **b)** Possible relationships between the three sibling species of the “*saxatilis*” complex, suggested by analyses of different molecular markers.

The snail genus *Littorina* provides ample opportunities for genomic studies of divergence, adaptive radiation and ecotype formation. In the North-Atlantic region, there is one planktotrophic species *L. littorea* and the five non-planktotrophic species of the subgenus *Neritrema*: *L. fabalis. L obtusata*, *L. saxatilis*, *L. arcana* and *L. compressa* [25]. The evolutionary history of these five species includes a split between two sister clades, 2 to 4 Mya, one containing *L. fabalis* and *L. obtusata* and the other containing *L. saxatilis*, *L. arcana* and *L. compressa* [25, 26]. Divergence of the sister-species *L. fabalis* and *L. obtusata* and between the three sibling species *L. arcana, L. compressa* and *L. saxatilis* is even more recent, 1.7 – 0.06 Mya by different estimates (Figure 1a,b). In both clades, studies of mtDNA variation revealed lack of reciprocal monophyly and shared alleles, likely due to incomplete lineage sorting [27–29]. However, shared haplotypes may also indicate rare hybridization between species, as has been suggested for *L. saxatilis* and *L. arcana* [30].

The relationships between the three sibling species *L. saxatilis*, *L. arcana* and *L. compressa* are especially interesting. All three species live in sympatry over large parts of their distribution and are morphologically very similar although *L. compressa* often can be distinguished by a characteristic shell pattern [25]. Species identification of *L. saxatilis* and *L. arcana* is possible only in the case of mature females, which have a jelly gland in egg-laying *L. arcana* and a brood pouch with embryos in ovoviviparous *L. saxatilis* [25]. A DNA marker has been suggested for discrimination of these two species, but 12-14% of analyzed individuals deviate from species-specific amplification patterns [30]. Notably, comprehensive phylogenies reconstructed from morphology and several types of genetic markers place either *L. arcana* and *L. compressa* or *L. saxatilis* and *L. arcana* as sister taxa (see Figure 1b), and hitherto this trichotomy has remained unresolved [26, 31].

All species of the subgenus *Neritrema* are polymorphic [25], but the most pronounced example of intraspecific variation is found in *L. saxatilis.* In particular, distinct “Crab” and “Wave” ecotypes have evolved in parapatric microhabitats as adaptation to crab predation or wave exposure [32]. Pairs of these ecotypes have been extensively studied in Britain, on the Galician coast of Spain, and on the west coast of Sweden to address mechanisms of ecotype formation and incipient speciation (reviewed in [33, 34]). The phylogeographic reconstruction of the species’ history based on mtDNA data suggests a close phylogenetic relationship through shared glacial refugia between British and Swedish populations, and a history of long isolation for Galician populations [28, 29]. A recent comprehensive study based on three nuclear introns, mtDNA and AFLP data showed that ecotypes most likely have evolved independently in the three regions as a result of local adaptations in the face of gene flow [35].

Thus Crab and Wave ecotypes of *L. saxatilis* present an opportunity to study mechanisms and genes involved in local adaptation and in the evolution of reproductive barriers in parallel systems. The genomic architecture of the adaptive variation in *L. saxatilis* is hitherto unknown, but transcriptome sequencing of the British ecotypes indicated a number of SNP’s associated with the ecotypes [36]. A genome scan of the British ecotypes using amplified fragment length polymorphism (AFLP) markers also revealed several outlying loci [37]. Two of them have been further characterized through sequencing of large genomic fragments around the outliers from a bacterial artificial chromosome library (BAC), but candidate genes could not be identified [38].

The application of genomic approaches in *Littorina* snails is needed to clarify phylogenetic relationships between closely related species and populations that have so far been based on one or a few loci. Furthermore, comprehensive genome-wide studies will be necessary for identification of genes and genome regions that are under diversifying selection and/or involved in recent or on-going speciation events. One way to search for genomic divergence between species and populations is by using array comparative genomic hybridization (aCGH), which is based on hybridization of labeled genomic DNA fragments to a microarray, representing a subset of the genome of the target species. aCGH has been widely used in model organisms and in human medical genetics [39]. Depending on the array design and platform used, aCGH can target different types of genomic variation – from chromosomal rearrangements and copy number variants (CNVs) to single nucleotide polymorphisms (SNPs) [40–42]. Probe design varies depending on the targeted type of variation, e.g. reliable SNP detection requires short (≤50 nt) probes with relatively low GC content and multiple tiling probes per target SNP [43–45]. On the contrary, oligonucleotide probes in a gene expression array are usually longer (50 -100 nt) and have higher GC content in order to tolerate single mismatches and provide reliable estimates of gene expression despite variation in DNA sequence among individuals [45–48].

aCGH has been used to detect gene loss and acquisition and highly polymorphic genes in bacteria [49], CNVs between different cultivars of rice [50] and for genotyping known SNPs in *Caenorhabditis elegans* [44]. Since aCGH design requires the reference genome, this method has not been available for non-model organisms. However, for species with existing transcriptome libraries and gene expression arrays, genomic DNA can be hybridized using transcriptome arrays to detect CNVs (by hybridization signal well above the average) and, to some extent, sequence divergence in coding parts of the genome (by an hybridization signal below the average). This approach has been applied successfully to identify rapidly evolving genes and CNVs in different strains of the ectomycorrhizal fungus *Paxillus involutus* [51, 52].

In the present study, we applied aCGH analysis to explore genome divergence at different evolutionary scales: species that are easily recognized by morphological characters and show reciprocal monophyly in traditional single-gene phylogenies (*L. fabalis vs.* three other *Littorina* species); recently diverged sibling species with unclear phylogenetic relationship *(L. saxatilis, L. arcana* and *L. compressa)*, geographically distant populations of a species with high level of population structure (*L. saxatilis*) and, finally, *L. saxatilis* ecotypes that have evolved repeatedly in the three different regions. The goals of the study are to detect overall patterns of genome divergence (how many genes show divergence at different evolutionary scales; how these numbers correspond to previous single-gene phylogenies; whether the same genes show elevated rates of evolution in different species) and to identify outliers for future studies.

For the experiments, we used a high-density oligonucleotide gene-expression array for *L. saxatilis*, representing more than 25,000 partial transcripts. To our knowledge, expression oligoarrays have not been used for aCGH before and the sensitivity of this method is unknown. The obvious advantage of this approach is that the array represents the coding part of the genome. The pitfalls are that intron/exon boundaries within the probes may have a large effect and we need to distinguish CNV’s from sequence divergence. To investigate this, we included in the array design the genomic sequences that are available for the species (see below) and mitochondrial DNA probes with mismatches. At the time of data analyses, we had produced the first preliminary draft of the *L. saxatilis* genome, which gave us an opportunity to explore in more detail what types of genetic variation was detected by our method. Specifically, we address the following questions:

1. What type of genetic variation can be detected by hybridization of genomic DNA to oligonucleotide transcriptomic arrays?
2. Does aCGH analysis confirm phylogenetic relationships among and within closely related species inferred from analyses of only a few genes?
3. Can we resolve the trichotomy between the three sister species *L. arcana*, *L. compressa* and *L. saxatilis*?
4. Does aCGH analysis confirm the high divergence between Galician and the two more northern populations of *L. saxatilis*?
5. Can we use this approach to detect adaptive variation underlying Crab and Wave ecotypes of *L. saxatilis* and test for parallelism of adaptations?
6. Are there genes with elevated rates of evolution in this lineage of *Littorina* and, if so, are similar or different genes involved in different species pairs?

## Methods

### Array design and genome information represented

We used an oligonucleotide microarray platform for *L. saxatilis* that was developed by NimbleGen Roche (090824_L_saxatilis_expr_HX12, 12X135K array format) and contained sequence information based on 25,205 partial transcripts, hereafter referred to as “genes”, from The Littorina Sequence Database, LSD [53, 54]. These transcripts were obtained mainly by 454 sequencing (454/Roche) of cDNA libraries from pooled tissues and individuals of British Crab and Wave ecotypes of *L. saxatilis* (see [54] for details). In addition to sequence information on transcripts, we added *L. saxatilis* genome sequences that were available in public databases at the time of the array design: 577,000 nt in total from four sequenced BAC clones: CH317-88D12, -123M16, -148L122 and -10N19 [38] [GenBank:CT476813, GenBank:CT757510, GenBank:CR974470, GenBank:CT027673]. This library was constructed using genomic DNA of four Crab-ecotype individuals from one British population (Thornwick Bay). In the array design, these BAC clones were divided into 578 fragments of 1,000 nt each, which for simplicity are also referred to as “genes”, although most of these fragments constitute non-coding DNA [38]. An 8,022 nt long mt genome sequence of *L. saxatilis,* also from a British population [55] [Genbank: AJ132137] was divided into 16 “genes” of 500 nt each and these were also included on the array, along with 14 transcripts from other mollusks and 19 flanking regions of microsatellites, developed for *L. saxatilis* [56] and *L. subrotundata* [57] and earlier used in population studies of *L. saxatilis* [58, 59].

Each “gene” was represented on the array by five non-overlapping 60-nt probes, except for transcripts with the total length <300 bp, for which probes overlapped. To provide optimal hybridization, probes were designed with 44% GC content, unless the whole fragment had lower GC content. In total, each array contained approximately 135,000 probes representing 25,835 “genes”, and each slide contained 12 identical subarrays.

### Sample collection, preparation and hybridization to microarray

*Littorina fabalis* were collected in the vicinity of the Tjärnö marine research station (University of Gothenburg). *Littorina arcana* and *L. compressa* were obtained from the east coast of Britain; since the males cannot be distinguished from *L. saxatilis*, we used only mature females of these species. For each of these species, four snails were included in the experiment (Table 1). *Littorina saxatilis* samples were obtained from three regions (Britain, Sweden and Spain) as pairs of local Crab and Wave ecotypes (Table 1). For each group (region X ecotype), we used four randomly chosen individuals; in total 24 individuals of *L. saxatilis*. Thus, the whole experiment included 36 snails for which genomic DNA extracts were individually hybridized to the array.

**Table 1:** ***Littorina*** **samples used for aCGH (n = 4 for each group)**

Species	Geographic region	Location name	Ecotype
*L. fabalis*	West coast of Sweden	Saltö	-
*L. compressa*	East coast of Britain	Black Rock	-
*L. arcana*	East coast of Britain	Great Castle Head	-
*L. saxatilis*	East coast of Britain	Thornwick Bay	Wave (H)
*L. saxatilis*	East coast of Britain	Thornwick Bay	Crab (M)
*L. saxatilis*	Galician coast, Spain	Baiona	Wave (SU)
*L. saxatilis*	Galician coast, Spain	Baiona	Crab (RB)
*L. saxatilis*	West coast of Sweden	Saltö	Wave (E)
*L. saxatilis*	West coast of Sweden	Saltö	Crab (S)

Genomic DNA was extracted from foot muscle tissue using a CTAB extraction method modified from [60] to include RNAase treatment and to increase DNA yield (protocol available upon request). DNA concentration and purity were assessed using a NanoDrop spectrophotometer (Thermo Scientific) and agarose-gel electrophoresis. For each group (species, population or ecotype, see Table 1) two individual DNA samples were labeled with Cy3 and two with the Cy5 dye. Labeling was performed with a starting amount of 1 μg of genomic DNA per sample, 5′-Cy random primers and Klenow fragments (NimbleGen/Roche Dual label kit), following the manufacturer’s protocol.

Individuals from the different groups were randomly distributed between the subarrays, i.e. each subarray hosted two individual samples, one labeled with Cy5 and one with Cy3; no common reference sample was included in the experiment. This experimental design was chosen in order to conduct intensity-based analyses of data instead of ratio-based analyses. Ratio-based analysis for dual-colour microarrays has been used commonly to control for the high inter-array variation in earlier microarray platforms. However, in high density synthetic oligoarrays the inter-array variance is much lower [61], which removes the need for reference sample and allows the use of intensity data from separate channels [62]. This design also helps to separate more reliably the types of genetic variation behind the low-hybridization signals in the absence of a reference genome. For example, in the reference design, low sample-to-reference ratios can be due to high sequence divergence in the sample or higher number of copies in the reference. Using normalized signal intensities instead of ratios, multiple-copy regions can be detected in all samples, as having signals twice or above the average, single-copy level.

For hybridization, 20 μg of Cy3- and Cy5-labeled DNA of two samples for each subarray were combined, vacuum-dried and resuspended in 12 μl of hybridization solution, of which 6 μl was applied onto a subarray for hybridization. Hybridization was performed in a NimbleGen Hybridization System at 42° for 48 hours following the manufacturer’s protocol (NimbleGen/Roche). After hybridization the slides were washed using the NimbleGen Washing kit and immediately scanned at 2-μm resolution using an Agilent G2565AA microarray scanner (Agilent Technologies, Santa Clara, CA).

### Image processing and data normalization

The array images were processed using the NimbleScan v.2.5 software (NimbleGen/Roche). First, we assessed the quality of the images according to the manufacturer’s guidelines and discarded images with signal intensity or other metrics outside the recommended range. (For those samples, we performed new labeling reactions and conducted hybridization on an additional slide; in total three slides were used in the experiment). After quality control, the signal intensity data for each channel were corrected for the local background signal, log2-transformed and used for normalization.

Normalization of microarray data is necessary to remove differences in signal intensity between individual slides and subarrays as well as the systematic difference in signal intensities of the Cy3 and Cy5 dyes. There are numerous algorithms for data normalization serving this purpose, including the Robust Multi-Array normalization algorithm (RMA; [63]), implemented in the NimbleScan software. RMA adjusts the raw signal data using a quantile method so that signal intensity data for all individual samples have similar normal distributions [64]. The assumption of similar signal intensity distributions in all samples is likely to hold for gene expression data sets, when many genes are expressed at similar levels in all samples and there are roughly equal numbers of sample-specific up- and down-regulated genes (though there may be exceptions, see [65]). For expression data conforming to this general pattern, RMA has been shown to perform very well [64]. For our data, however, based on hybridizations of genomic DNA to a transcriptomic array, we did not know *a priori* whether the assumption of similar signal intensity distributions would be met. In theory, heterologous hybridizations (i.e. when a DNA sample from one species is hybridized to an array developed for another species) may produce signal intensity distributions skewed towards low values, if interspecific sequence divergence is large enough to lower hybridization efficiency in many genes.

To test this, we compared the normalization by the RMA procedure in NimbleScan to a method that is not based on the assumption of similar signal intensity distributions in all samples. For this, we used an ANOVA normalization, i.e. fitting the “normalization” ANOVA model, that estimates non-biological variation due to Dye [Fixed] and Subarray [Random] and saving the residuals [66]. Normalization was carried out for the effect of the subarray and not of the array since there was no systematic difference between the three slides (arrays) used in the experiment, although we did observe significant variation in signal strength between subarrays within each slide. The interaction term Dye*Subarray was not included because it defines a single sample in the experiment and thus is confounded with levels of biological variation (between species and populations).

In all samples, both normalization methods produced signal intensity distributions of similar shape: slightly bimodal with a second right peak of high signal and with a left tail with the low-signal data (Additional file 1: Figure S1 shows an example of the two distributions in one sample). Moreover, there was a high correlation between RMA-normalized and ANOVA-normalized signal per gene (*R*^*2*^ ≥ 0.95 in all individual samples, *p* < 0.00001). Thus, we concluded that both normalization methods performed similarly on our dataset and used the RMA-normalized data in the subsequent analyses.

Usually, in the second step of the RMA algorithm, the signal intensities for individual probes (n = 5 per gene) are summarized to obtain a single value for each gene using the method of Irizarry *et al.* [63]. This step is based on the assumption that, in gene expression data, the true signal intensity level for all probes, representing one gene, should be the same. In aCGH data, however, hybridization efficiency for individual probes depends on sequence similarity between them and the hybridized DNA and thus can vary between probes that come from different fragments of one gene. For this reason we performed both gene-level and probe-level data analyses.

### Statistical analyses of genomic divergence between species and populations

To test whether genome divergence between *L. saxatilis* and the other studied *Littorina* species lowered the success of heterologous (interspecific) DNA hybridization, the variation of average log2-signal intensities between the samples was analyzed by fitting a mixed analysis of variance (ANOVA) model with Species [Fixed] + Dye [Fixed] + Subarray [Random] using the JMP 10.0.0 software (SAS Institute, Inc.). All three factors had highly significant effects (*p* < 0.0001), and signal intensity levels were compared between the species using Student’s *t*-test on residuals after the effects of Dye and Subarray had been removed.

Principal component analysis (PCA) using the Qlucore Omics Explorer 2.1 (Qlucore AB, Lund, Sweden) was applied to visualize the variation in hybridization success between species and populations (treating each gene as a variable). PCA across all variables is useful to detect very strong patterns in the dataset, but many individual effects are likely to be obscured by the high total variation in a dataset with very many variables (25,801 variables in this case). Hence, to further explore patterns in the data, PCA was also performed including only genes that showed significant differences in hybridization signal intensities between the groups. These genes were identified by one-way ANOVA for each genes and applying a false-discovery rate q = 0.05 cut off across the tests [67]. Signal intensity data for the genes with significant variation in the species pairs were used to produce a heat map (representing the strength of hybridization signal in different samples) using the Qlucore Omics Explorer. In addition, we performed hierarchical clustering analyses of different species and populations based on hybridization signal intensity in all genes and with 20,000 bootstrap permutations of the data. This was done using Euclidean distance and the single linkage clustering algorithm in the *maanova* package [68] in R [69].

Finally, we identified genes with significant differences in hybridization success for pairs of species and for each sample against the British *L. saxatilis* sample (since both the BAC library and most of the transcript libraries, used for the array design, were based on the British *L. saxatilis* ecotypes) by performing *t*-tests for each gene and setting a cut-off at q = 0.05 in the Qlucore Omics Explorer. The number of significant genes in pair-wise comparisons was used for neighbour-joining clustering of species and populations in the package *APE* v.3.0.7 [70] in R [69]. To test whether the same genes show elevated divergence rates between different species, lists of pair-wise significant genes were compared using Venn diagrams using the BioVenn tool [71].

### Sensitivity analysis and identification of candidate duplicated genes

For single-copy genes, divergence between the hybridized DNA sample and probes on the array results in a lower hybridization signal. Since a majority of the probes were designed from cDNA sequence information, we expect that some probes will span exon-intron boundaries in the genome. In such cases only a part of the labeled genomic DNA fragment is complementary to the probe and this should significantly reduce hybridization. In addition, mismatches at the nucleotide level between the probe on the array and the hybridized DNA can decrease the hybridization efficiency. One mismatch per 60 nt (probe length) is hardly detected [41], but several mismatches per probe are likely to have a negative effect on the hybridization [43]. In addition to the number of mismatches, other factors, such as type of base changes between the probe and the hybridized DNA, their position within the probe and the GC content of the probe have been shown to have large effects on hybridization success for long-oligonucleotide arrays [41, 43].

To investigate what types of genomic variation were detected by our aCGH-method, we used two approaches: comparing sequences of the array probes with low and high signal intensities to the draft genome of *L. saxatilis* and re-sequencing fragments of the mitochondrial *cytochrome b* gene, represented on the array, in the analyzed individuals. The *Littorina saxatilis* genome sequencing project is currently being carried out by the Linneaus Centre for Marine Evolutionary Biology at Gothenburg University [72]. At the time of the data analyses presented here, the available assembly was performed on 101 Gbp of Illumina reads from a 300 nt-insert library using the CLC Assembly Cell v 4.0.6, and produced a total assembly size of 473 Mbp and N50 contig size of 916 nt. Sequenced genomic DNA comes from a single individual of the Swedish Crab-ecotype of *L. saxatilis* from the island Saltö, which is the same population as included in the present CGH experiment. While there is certainly much genetic variation within any population, many protein-coding sequences are likely to be invariable. Given the large amount of cDNA sequence information on our array we assumed that the genome sequences of the snails in our aCGH experiment were basically the same as in the genome assembly. Hence, we correlated differences between probes on the array (representing the British *L. saxatilis* populations) and the genome sequences (representing the Swedish *L. saxatilis* Crab-ecotypes) with the hybridization signal intensities of the Swedish *L. saxatilis* Crab-ecotypes. Genome contigs corresponding to different probes from the array were identified as the top hit using BLASTN 2.2.25+ algorithm [73]. We calculated correlations between hybridization signal intensities and BLASTN top hit parameters (query match length, identity and number of mismatches) as well as with GC content of the probes in JMP 10.0.0.

For the mitochondrial *cytochrome b* gene fragments, we included several sequence variants on the array representing previously detected variants of this gene in *Littorina* [29]. We amplified and sequenced the *cytochrome b* fragment (as described in [29]) in 34 of the 36 individuals used in the aCGH, counted the number of actual mismatches between the obtained sequences and array probes, and compared the hybridization signal intensities between probes with different numbers of mismatches. The haploid mtDNA fragment was chosen for this sensitivity analysis in order to avoid potential heterozygotes.

**Figure 2:**
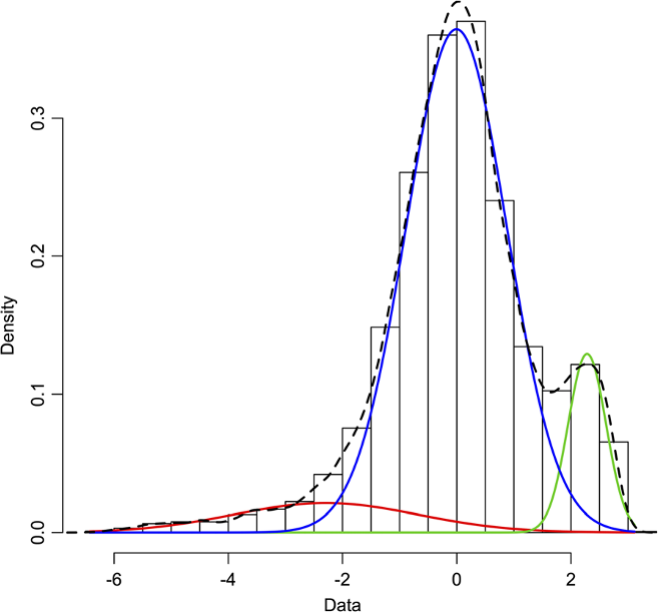
**aCGH hybridization signals in** ***Littorina saxatilis*** **(average of 24 individuals).** Frequency histogram shows distribution of log-2 normalized and centred signals (zero corresponds to overall signal average); colour curves – a fitted mixture of three normal distributions from *mixtools* R package. Red curve – low signals, blue curve – normal signals (single-copy genes), green curve – high signals due to multiple gene copies.

Finally, array sequences representing duplicated genes and multiple-copy variants are expected to produce two-fold or higher hybridization signals than single-copy variants. To detect such genes we fitted a model with a mixture of three normal distributions, representing average-signal genes, low-signal outliers (for example, due to exon-intron boundaries, see above) and high-signal outliers (candidate multiple-copy genes) to signal distributions in hybridized samples. This was done using the EM algorithm in the *mixtools* package [74] for R [69]. High-signal outliers were defined as genes showing signal levels greater than or equal to the mean minus two standard deviations of the right hand peak (Figure 2). Lists of candidate genes were obtained for each of the groups (species, population, ecotypes) and the lists were compared between the groups. In the data on the Swedish *L. saxatilis* Crab-ecotype, we calculated sequencing coverage of genome contigs, containing genes with the normal signal level and compared it to sequencing coverage of genome contigs containing genes with high signal level (likely to be present in multiple copies in the genome).

## Results

### Genes with low hybridization signal

In order to conduct genomic comparisons of populations and species we first needed to understand what type of genome variation was causing the variation in hybridization signal. To do this, we compared sequences on the array that showed low hybridization signal in the Swedish Crab ecotype to the genome sequences from a preliminary *L. saxatilis* genome assembly made from an individual of the same ecotype and from the same locality. In these analyses we assume that the genomes of the sequenced individual and of the snails used for the aCGH experiment are identical.

The log2-transformed signal intensity per gene, averaged over the four snails of the Swedish *L. saxatilis* Crab-ecotype, had an average of 13.03 ± 1.34 (±standard deviation (SD)). We defined low hybridization signal to be below 10.35 (i.e. 2 × SD below the average), which corresponds to a drop of more than six-times in the fluorescent intensity on an absolute scale. We chose this method instead of using parameters of a left-side distribution from the mixed-distribution model since the left peak was not clearly defined and largely overlapped with the main peak (Figure 2). This resulted in 973 “genes” on the array of which 137 came from the CH317-123M16 BAC-clone, 828 came from *L. saxatilis* transcripts, seven from heterologous sequences and a random class of oligonucleotides with 44%-GC content that are included on NimbleGen oligoarrays as standard procedure.

**Figure 3:**
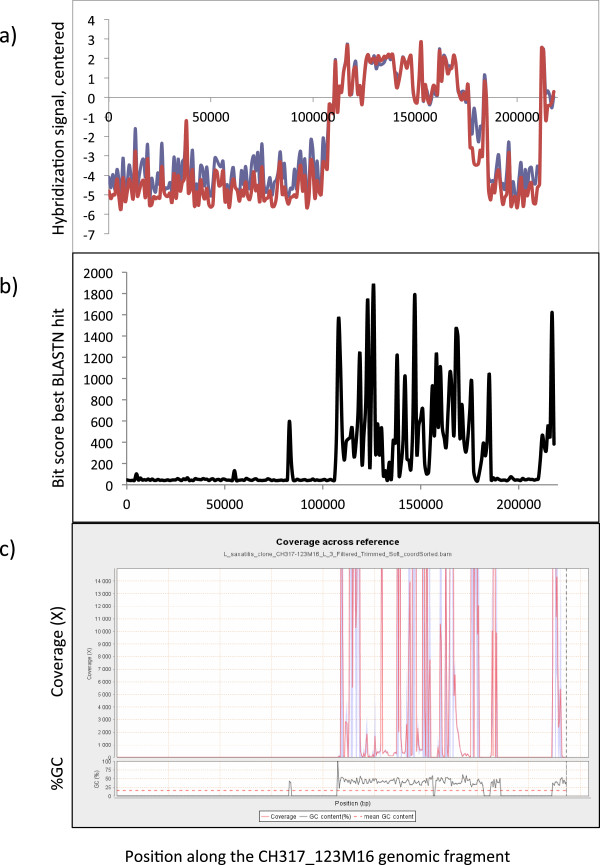
**Low aCGH signal for CH317_123M16 fragments suggests deletions in** ***Littorina saxatilis*** **genome. a)** Centered hybridization signals in Crab ecotypes of *L. saxatilis* from Sweden (red line) and Britain (blue line) calculated for 1,000-nt intervals of CH317_123M16 BAC. **b)** Results of BLASTN search in *L. saxatilis* genome assembly for 1,000-nt intervals of CH317_123M16 BAC, represented by a bit score of the top hit. **c)** Mapping of reads from the Illumina sequencing of 300-nt genomic library to CH317_123M16 BAC.

The CH317-123M16 BAC-fragment [GenBank:CT757510] has a total length of 218,205 nt and was divided into 218 fragments (“genes”), each 1,000-nt long, on the array. Inspection of data for all fragments of this BAC clone showed that hybridization success in Crab ecotype-snails varied along the BAC clone, with two regions of very low hybridization signal (1 – 108,000 nt and 186,000 – 211,000 nt) and two regions of average or high hybridization signals (108,000 – 186,000 nt and 211,000 – 218,205 nt); see Figure 3a, red line. We performed BLASTN searches for the 1,000-nt fragments of this BAC-clone against the *L. saxatilis* draft genome assembly (in order to obtain all matches, word size was set to seven and filters for repeats and low information content were switched off). The fragments with low hybridization signals had only short matches to the genome sequence that were likely to occur by chance while the fragments with high hybridization success had long matches with high similarity to the genome data, as represented by alignment bits scores in Figure 3b. Overall, the distribution of alignment bit score along the BAC clone closely followed hybridization success (Figure 3b). However, the genome draft assembly at present has a total size of 473 Mbp, while the haploid genome size estimated by flow cytometry is 1.3 Gbp [75], indicating that some regions are problematic to assemble from short reads and are missing in the assembly. To test whether this was the reason why we did not find some BAC fragments in the genome assembly, we mapped Illumina reads from the genome sequencing to the CH317-123M16 BAC clone using Qualimap [76]. However, the regions of the BAC clone that were not found by BLASTN also had zero coverage by unassembled reads (Figure 3c).

Thus, it is likely that two large fragments of the CH317-123M16 genomic region are absent in the genome of the Swedish Crab-ecotype snails that were used in our aCGH experiment as well as in the genome of another Crab-ecotype individual that is being sequenced in the genome project. The CH317-123M16 BAC clone was originally characterized for the British Crab-ecotype [38]. Surprisingly, the hybridization success along CH317-123M16 for four snails of the British Crab-ecotype were very similar to the one described for the Swedish Crab-snail individuals (Figure 3a, blue line). This suggests that there may be an insertion-deletion polymorphism for large genomic regions in *L. saxatilis*, and the deletion variant may be rather common. Alternatively, there may be an artifact in the BAC assembly.

**Figure 4:**
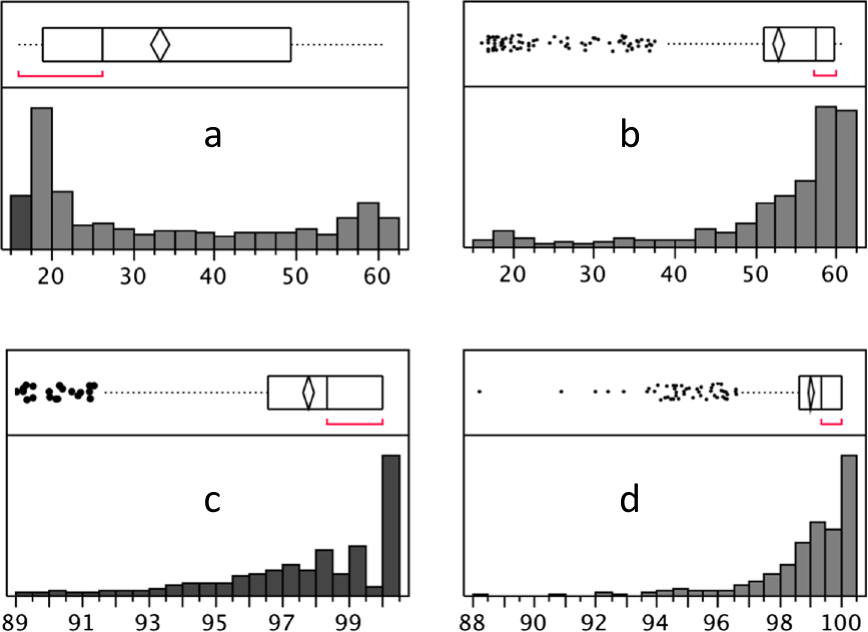
**Exon/intron boundaries cause low hybridization to 60-nt probes in** ***Littorina*** **aCGH.** Sequences of aCGH probes with low **(a, c)** and normal **(b, d)** signal in the Swedish Crab ecotype of *L. saxatilis* were compared to the *Littorina* genome draft by BLASTN. Low-signal probes had shorter lengths of the top hit than normal-signal probes **(a** ***vs.*** **b)**, indicating exon/intron boundaries within low-signal probes. Identity scores for low-signal probes were slightly lower than for normal-signal probes **(c** ***vs.*** **d)**. Each distribution is shown as a frequency histogram and a box plot (top panels), showing 25% and 75% quantiles (box), mean ± C.I. (diamond), median (vertical line), outliers (dots) and the densest region of the distribution (red bracket).

Low hybridization success of genomic DNA from the Swedish *L. saxatilis* Crab-ecotype snails was also observed for 828 “genes” on the array representing transcripts. For these, we retrieved probe sequences (five for each transcript) and performed BLASTN search in the *Littorina* genome assembly with default BLASTN parameters. For comparison, we performed the same analyses for the transcripts that had hybridization signal intensity around the average. Probes representing four of the low-signal transcripts were not found in the genome; the rest of the low-signal probes showed only partial similarity to the genome contigs (Figure 4a, median hit length = 26 nt while probe length = 60 nt), although often with high identity (Figure 4c; median identity = 98.3%). The majority of the probes yielding average-signal transcripts had full-length or nearly full length-matches to contigs from the genome assembly (Figure 4b, median hit length = 57 nt) and identity close to 100% (Figure 4d, median identity = 99.3%). Since the probes were derived from transcript sequences, partial matches with high similarity to the genome contigs are expected when a particular probe spanned the boundary between two exons. In such cases, 5′ or 3′ ends of hybridized genomic DNA fragments contained an intron sequence and did not match the probe. Analyses of the BLASTN results for probes with partial similarity to the genome (hit length < 50 nt, 3276 probes) showed that in most cases (3,131 probes) the second BLASTN hit, including the other (5′ or 3′) end of the probe, was on a different scaffold. In a few cases (145 probes), the second BLASTN hit was on the same scaffold. This result is expected given that the genome scaffolds at present are very short (N50 = 916 nt) and consecutive exons are likely to be on different scaffolds. Thus, the transcripts with lower hybridization success seem to be the ones where all or a majority of the probes happened to span exon boundaries.

However, some of the low-signal probes (642 out of 4,176, see Figure 4a) had full-length matches to the genome contigs. For those we calculated the GC content and the number of mismatches to the genome sequence and compared them to the same parameters for 2,520 probes with average hybridization signal and full-length match to the genome sequences. While the average number of mismatches in the low-signal probes was only slightly higher compared to the average-signal probes (1.1 *vs.* 0.4 nt out of 60*, t*-test *p* < 0.0001), the GC-content of the low-signal probes was low compared to the average-signal probes (35.1% *vs.* 43.8%, *t*-test *p* < 0.0001). The optimal GC-content for NimbleGen oligoarray-probes is 44%, and average-signal probes were all close to this value. However, due to variation in GC-content of partial transcripts used in the array design, some probes had a lower, i.e. sub-optimal, GC-content and this appears to have had a large negative effect on hybridization.

### Effect of mismatches on the hybridization signal

As shown above, genes with very low hybridization efficiency in the Swedish Crab-ecotype of *L. saxatilis* depended on occurrences of exon-intron boundaries within the probe sequences or low GC-content of the probes. To further investigate the sensitivity in hybridization of 60-nt probes to sequence divergence (i.e. occurrence of one or more substitutions between hybridized DNA fragment and the probe), we looked at the correlation between signal intensity and the number of substitutions for the probes that had full-length matches to the genome sequences (excluding the probes coming from putatively duplicated genes, see below). We identified 68,898 such probes with 0 up to 10 nt differences compared to the genome sequence. Among these the hybridization signal intensity did not decrease with the number of substitutions (R = 0.02, p = 0.0002) but correlated positively with the GC content of the probe (R = 0.42, p < 0.0001).

**Figure 5:**
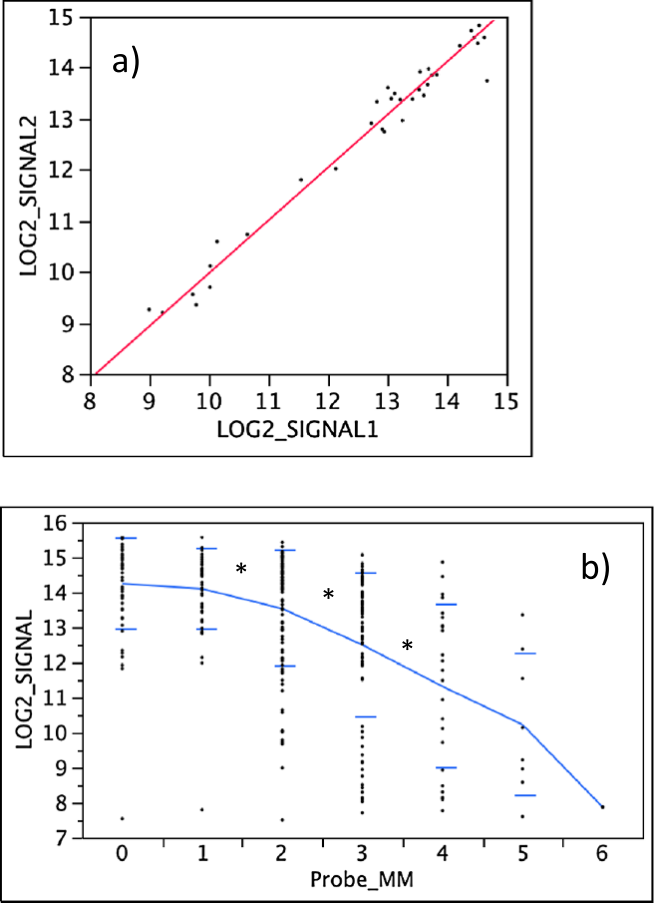
**Effect of mismatches on aCGH signal in** ***Littorina*** **in re-sequenced** ***cytochrome b*** **probes.** A fragment of the mitochondrial cytochrome b gene was re-sequenced in 34 *Littorina* individuals and compared to the probe sequences to identify mismatches. **a)** Signals from two replicates of identical probes; **b)** The effect of 1-6 mismatches within the probe on the hybridization signal (blue lines show average and SD for each class; asterisks denote significant decrease in signal between the two levels, t-test, p < 0.05).

In the analyses above we did not know the actual genome sequences of the individual snails used for hybridization, although we expected that they were highly similar to the genome of the sequenced Swedish Crab-ecotype individual, originating from exactly the same locality. To test directly how the number of mismatches affected the hybridization, we sequenced a fragment of the mt *cytochrome b* gene for 34 out of 36 individuals used in our experiment and calculated the number of mismatches between these sequences and the *cytochrome b* probes on the array. For this gene, the array contained two sets of probes designed from two non-overlapping fragments, and each set contained five sequence variants that differed from each other by 1-4 SNPs, making a total of 10 probes. In addition, five of these probes were included on the array twice. For replicated probes, there was a high consistency of hybridization for identical probe replicates (Figure 5a, R^2^ = 0.99, p < 0001). The direct sequencing of these fragments in the DNA samples revealed up to six mismatches between the probe and hybridized DNA. Single mismatches did not affect the success of hybridization (Figure 5b, *t*-test *p* > 0.05) while two or more mismatches significantly decreased the hybridization efficiency (Figure 5b; *t*-test *p* < 0.05). However, there was high variation in hybridization efficiency within each mismatch class of probes. This could not be explained by the length of the perfect match, the position of the first mismatch relative the 5′ end of the probe or by change in the GC content caused by the mismatches (tested by linear correlations, p > 0.05). However, probes with lower GC content (28-35%) were more sensitive to mismatches than probes with higher GC content (40-48%). To test whether the level of variation in the mt *cytochrome b* gene is representative for other genes in the *Littorina* genome, we calculated the proportions of variable sites in the British population of *L. saxatilis* in the *cytochrome b* fragment using data from [29] and for the transcripts used in the array design based on data from [36]. The proportions were similar: 0.03 for *cytochrome b* and 0.01 on average for the transcripts.

Altogether our sensitivity analysis showed that occurrences of exon-intron boundaries and the GC-content of probes had major effects on the hybridization success when using genomic DNA for hybridization. Occurrence of two or more nt mismatches within the probe may decrease hybridization efficiency, but to a smaller extent, and this effect varied widely among the probes.

### Candidates for gene duplication

In addition to the genes with low hybridization signal intensities there were a number of genes showing hybridization signals far above the average, possibly resulting from segmental duplications in the genome. The overall signal intensity distribution for each sample could be described as a mixture of three normal distributions: a small low-signal peak of genes, a major peak in the center and a high-signal peak of genes with intensities well above the average. Figure 2 shows the mean signal intensity distribution for the 24 *L. saxatilis* individuals together with a fitted model; distributions for other species were similar and are not shown. Parameters for the fitted distributions for each species are presented in Table 2. After fitting the model, we defined high-signal genes as those with intensities greater than or equal to the mean minus two standard deviations of the right side distribution and normal-signal genes as those within two standard deviations of the mean for the central distribution. The cut-off at two standard deviations was chosen in order to include 95% of the distributions and at the same time avoid a large overlap between the thresholds (i.e. number of genes included in both groups).

**Table 2:** **Identification of potentially duplicated genes and genomic regions in** ***Littorina***

Species	m1 ± S.D.	m2 ± S.D.	m3 ± S.D.	N total dupl.	% of total	Overlap with ***L. saxatilis***, %	Transcripts	BAC regions	mtDNA	Other
*L. saxatilis*	−2.29 ± 1.62	−0.01 ± 0.88	**2.28 ± 0.33**	2573	10.0		2435	134 (incl.E10)	3	1 (Lsub16)
*L. compressa*	−2.64 ± 1.79	−0.2 ± 0.84	**1.85 ± 0.28**	2763	10.7	85.1	2618	142 (incl.E10)	2	1 (Lsub16)
*L. arcana*	−2.64 ± 2.02	0.21 ± 0.81	**2.22 ± 0.29**	2000	7.7	93.2	1903	93 (incl.E10)	3	1 (Lsub16)
*L. fabalis*	−2.43 ± 1.97	0.02 ± 0.93	**2.37 ± 0.38**	1756	6.8	92.8	1672	81 (incl.E10)	2	1 (Lsub16)

A model with three mixed normal distributions was fitted to overall distributions of hybridization signal intensity in each species: 1 – low signal genes, 2 – normal signal genes (single-copy), 3 – high signal (potentially multiple-copy) genes, in bold. See also colour curves in Figure 2. m ± S.D are means and standard deviations for three normal curves in each species.

A relatively high number of genes on the array, approx. 10%, showed a high hybridization signal and thus indicate multiple copies in the *Littorina* genomes (Table 2). For the Swedish Crab-ecotype of *L. saxatilis*, 3,315 genes belonged to the high-signal peak of the distribution, compared to the 20,122 genes in the middle peak, the normal-signal distribution (333 genes were included in both groups). For these two groups of genes, we first retrieved genome sequences from the *Littorina* genome assembly corresponding to these array sequences (based on the top BLASTN hit with E-value set at 1e-10). Of the genes with normal signal levels, 94% were found in the genome assembly producing 13,956 putatively single-copy genome contigs. Of genes showing high signals, 90% were found in the genome assembly producing 2,067 genome contigs that possibly contain segmental duplications. (Some transcripts and BAC fragments were mapped to the same genome contig). Second, we compared the genome sequencing coverage for these two groups. Median sequencing coverage for single-copy contigs was 50× (i.e. close to the calculated average sequencing coverage, 67×), while multiple-copy genome contigs had generally higher coverage with median at 880× (Additional file 2: Figure S2).

In the analysis of all *L. saxatilis* individuals together we identified 2,345 genes as potentially present in many copies. Of these only 133 showed similarity to known proteins, and mainly to reverse transcriptases found in various organisms (see Additional file 3: Table S1, annotations were taken from [45]). Reverse-transcriptase genes are known to be present in eukaryotic genomes in many copies as a part of retrotransposons and long interspersed elements (LINEs). Similarly, the E10 genomic fragment [GenBank: EF428423], coming from genome scans of the British ecotypes of *L. saxatilis* and containing signatures of SINE-retrotransposon elements [38], showed evidence of multiple copies in aCGH (Table 2). Thus, at least some of the segmental duplications in the *Littorina* genome suggested here by aCGH are likely to be associated with different types of repeats and transposable elements.

A more surprising finding is that a large proportion of genome regions available from a previously characterized *L. saxatilis* BAC-library suggest high copy numbers: 133 kb of 577 kb, or 23%, in *L. saxatilis*. (A list of transcripts and BAC regions with high hybridization signal in *L. saxatilis* is provided in Additional file 3: Table S1). In addition, three regions of mtDNA, containing *tRNA*s, *NADH-6* and *cytochrome b* genes showed high hybridization signals in all species. High hybridization signal for mitochondrial genes on the array is expected since the mitochondrial genome is present in more copies per cell than the nuclear genome. Additional inspection of signal data also revealed signal intensities that were two times the average for other mitochondrial genes (*COI*, *COII*, *ATPase-6*, *ATPas-8* and *NADH-1*). At the same time, mitochondrial regions containing genes for small and large subunits of ribosomal RNAs hybridized at a level of half or less, of the average, possibly due to a propensity of these DNA fragments to form secondary structures that impede hybridization.

### Genome divergence between four closely related *Littorina*species

**Figure 6:**
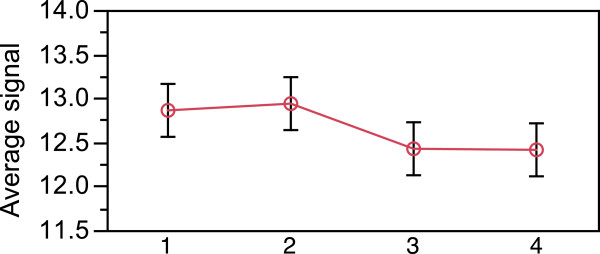
**Overall comparative genomic hybridization levels in four** ***Littorina*** **species.** 1 *– L. saxatilis,* 2 - *L. arcana,* 3 – *L. compressa,* 4 – *L. fabalis.* Bars show 95% confidence intervals.

Overall hybridization success (measured as average signal intensities after removing the effect of dye and subarray) varied among the species (ANOVA *p* < 0.001) and was significantly lower for DNA samples of *L. fabalis* and *L. compressa* than for DNA samples of *L. saxatilis* and *L. arcana* (*t*-test *p* < 0.05; Figure 6). However, the drop of signal was small, *e.g*. the differences in average hybridization signal intensity between *L. fabalis* and *L. saxatilis* was 0.45 on a log-2 scale, or 1.4 times on an absolute scale.

**Figure 7:**
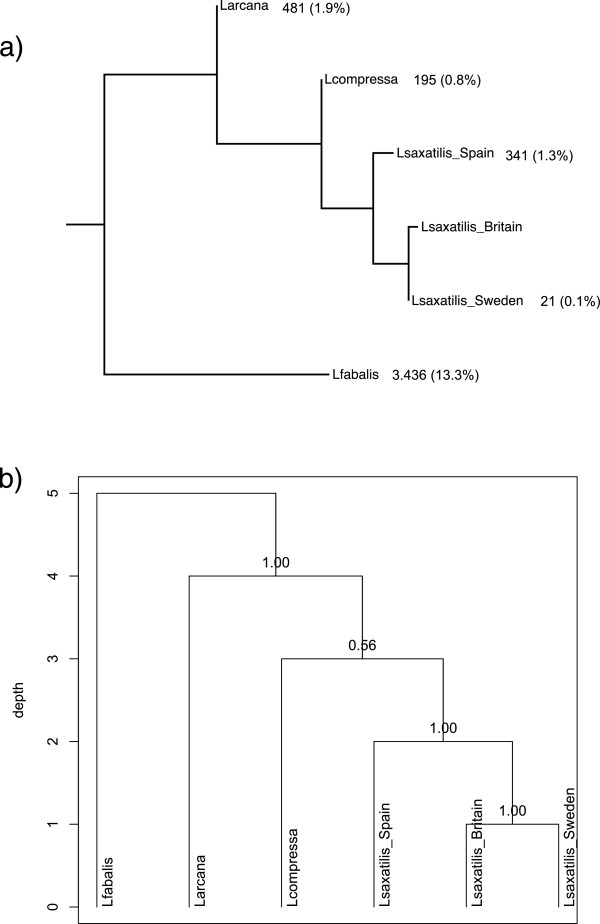
**Clustering of** ***Littorina*** **species and populations based on aCGH data. a)** Neighbour-joining clustering by the number of divergent genes between *Littorina* species. Genes with significantly different hybridization signal were identified in comparisons of species and populations against *L. saxatilis*-Britain (applied cut-off q = 0.05). Numbers of divergent genes and their percentage of the total number of genes on the array are shown along the branches. **b)** Consensus tree of 20,000 bootstrap replicates based on all genes on the array. Numbers showed proportion of trees containing each cluster.

Neighbour-joining clustering based on the number of genes that differed significantly in all pair-wise contrasts between the species and populations (Figure 7a) corroborated the phylogeny of this group (Figure 1a). Interestingly, differentiation between the British and Spanish populations of *L. saxatilis* in this analysis was of similar magnitude to that among the sibling species. Hierarchical clustering based on hybridization signal intensities from all genes on the array also produced a tree similar to the phylogeny of the group but the order of splits between *L.arcana*, *L. compressa* and *L. saxatilis* had low support and could not be resolved (Figure 7b).

**Figure 8:**
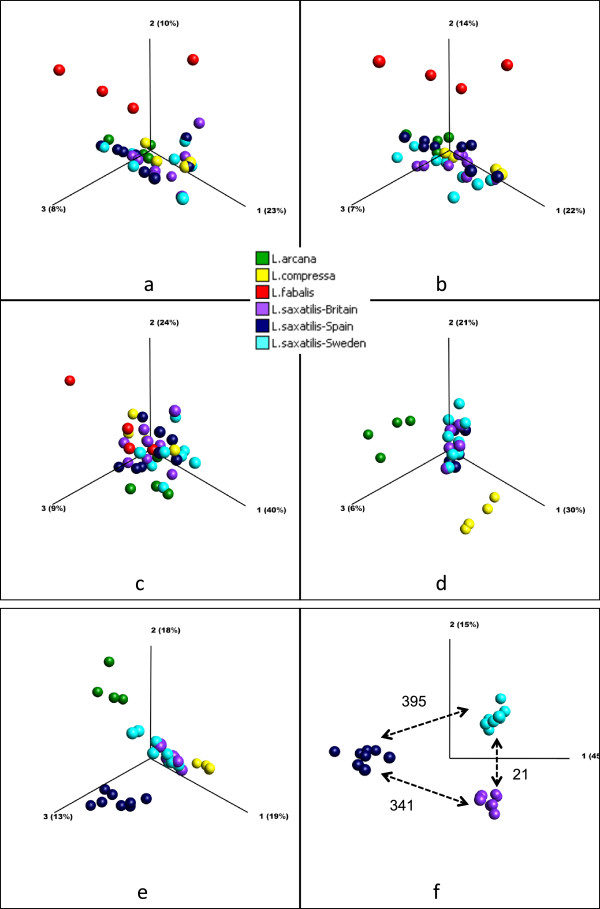
**PCA plots comparing genomic hybridizations of** ***Littorina*** **species. a)** Four species, all sequences (25,801 genes). **b)** Four species, only BAC fragments (580 genes). **c)** Four species, only mt DNA sequences (16 genes). **d)** Three sibling species, based on genes with significant variation among species (n = 1,094). **e)** Three sibling species and geographic populations of *L. saxatilis*, based on genes with significant variation among these groups (n = 2,232). **f)** Geographic populations of *L. saxatilis*, based on genes with significant variation among groups (n = 756). Also shown are numbers of significant genes for pairwise comparison of the populations.

In PCA plots based on signal intensity variation across all sequences on the array, *L. fabalis* was well separated from the other three species (Figure 8a). Similar results were obtained when we included only sequences representing transcripts (this plot was identical to the one including all genes, but is not shown), or including only BAC sequences, containing mainly non-coding regions (Figure 8b). PCA based on mitochondrial genes only, however, did not show separation between species, except for the outlying *L. fabalis* individuals (Figure 8c). This can be partly due to a low number of mitochondrial genes on the array (only 16, as compared to 25,205 genes for transcripts and 580 for BAC fragments) and partly due to the fact that divergence in mitochondrial gene sequences is mainly at the substitution level, which in the sensitivity analyses above was found to affect hybridization less than gene duplications and genomic deletions.

The three sibling species *L. saxatilis*, *L. arcana* and *L. compressa* did not show separation in PCA analysis based on all genes. However, after excluding the *L. fabalis* samples, and using only 1,094 genes that showed significant differences in hybridization among the three remaining species (per gene ANOVA, q = 0.05 level), *L. arcana*, *L. compressa* and *L. saxatilis* formed distinct groups, separated along the different axes (Figure 8d). To compare the degree of divergence between these sibling species with the intraspecific variation in *L. saxatilis*, we repeated the analysis with *L. saxatilis* divided into the three geographic regions. Notably, in this case the samples from the Spanish population of *L. saxatilis* also formed a distinct group, with the magnitude of separation close to that among the three sister species (Figure 8e).

### Variation in *L. saxatilis*due to geographic regions and ecotypes

In the analysis including only *L. saxatilis* individuals, we found genome divergence among the three geographic regions (Spain, Britain and Sweden) in 756 genes (per gene ANOVA, q = 0.05). In the PCA based on these genes, the Spanish population separated from the other two populations along the first axis explaining 45% of the variation, while the British and Swedish populations separated along the second axis explaining 15% of the variation (Figure 8f, the third axis is not shown in this plot since it explained only 6% of the variation).

No genes showed significant differences in hybridization between the ecotypes of *L. saxatilis*, neither in the comparison of Crab *vs*. Wave ecotypes across the three regions, nor in separate comparisons of the ecotypes within each region (per gene ANOVA, q = 0.05 level). However, we found some indication that there may be CNVs between the ecotypes across all the regions: there were 328 genes that showed evidence of multiple copies in one but not the other ecotype (Additional file 3: Table S1). Of these, only 17 had annotations in LSD and mainly to proteins containing the reverse-transcriptase domain (Additional file 3: Table S1).

### Genes with high divergence rates

Significant differences in aCGH signal intensities between the species indicate genes and genome regions with elevated divergence rates and/or CNVs. We identified such genes in different species and populations in comparison to the *L. saxatilis* samples from Britain, since the array design was based on sequence data from the British population. The highest number of genes that differed significantly from the British *L. saxatilis* was found in *L. fabalis*; the numbers in *L. arcana* and *L. compressa* were roughly an order of magnitude lower (Figure 7a).

**Figure 9:**
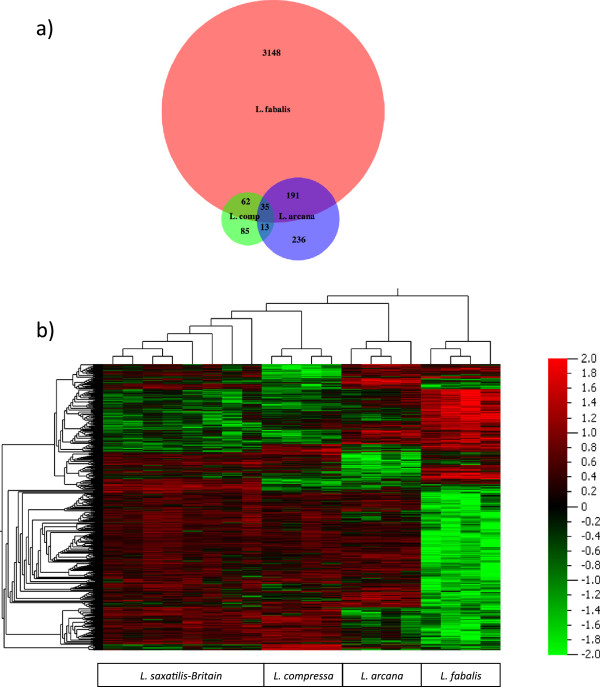
**Genes with high divergences between** ***Littorina*** **species. a)** Venn diagram showing number of genes with significant (q = 0.05) divergences between *L. saxatilis*-Britain and each of the three other species; **b)** Heatmap showing hybridization success in the four *Littorina* species in the genes with significant divergence between the species.

We compared cohorts of genes that showed significant differences between the British populations of *L. saxatilis* and one of the other three species (full lists are given in Additional file 4: Table S2). Of 3,469 genes, 266 were found in two comparisons and 35 showed divergence between *L. saxatilis* and all three other species (Figure 9a); most of the genes showed significance for only one pair of species. Similar results were obtained from a heat map of the signal strength for the 3,469 genes in different samples (with green colour corresponding to a signal intensity below average and red colour to a signal intensity above average: green and red clusters are mainly species-specific (Figure 9b)). For most of the genes with significant variation the hybridization efficiency was higher in *L. saxatilis* than in the compared species (indicated by green colour in Figure 8 and positive differences in Additional file 4: Table S2). However, a number of genes showed differences in the opposite direction, especially in *L. fabalis*, possibly due to higher copy numbers in this species compared to *L. saxatilis* or mutations increasing GC content of the sequences.

For different *Littorina* species, the high-signal portion of the distribution contained 1,756 – 2,763 genes (Table 2), which is approximately 10% of all sequences represented on the array. In general, there was a large overlap between the species suggesting that expansion of these genome regions occurred before the diversification of this littorinid lineage (Table 2).

## Discussion

In the present study we applied hybridization of genomic DNA from several species and populations of the North-Atlantic *Littorina* to an oligonucleotide array representing mainly *L. saxatilis* transcriptional sequence information. We showed that this approach can successfully detect CNVs, deletion and high sequence divergence (>1 substitution per 60 nt) but that it is not sensitive enough to detect single SNPs. Using this method we found a close agreement between patterns of genomic hybridization and previous phylogenetic reconstructions for this group based on only one or a few genes. However, we were not able to detect genes and genomic regions that have diverged between the Crab and Wave ecotypes of *L. saxatilis*. Below we discuss these results in detail.

### Types of genome divergence detected by genomic DNA hybridizations to oligonucleotide gene expression arrays

Our sensitivity analyses showed that hybridization of genomic DNA to long-oligonucleotide arrays designed mainly from transcript sequences can potentially detect sequence divergence at the level of roughly 2 or more mismatches per 60 nt probe length (or above 3% divergence). However, the signal drop could not be directly translated into the number of mismatches due to high variation between the probes. We observed a significant correlation between number of mismatches and hybridization signal intensity in the subset of mitochondrial probes, but not for all probes on the array. It has been shown that other factors, such as the probe GC content, the position and type of substitution, may have larger effects on hybridization success using oligonucleotide arrays than the number of mismatches [41, 43, 48]. This is in agreement with our results, where the most important factor for hybridization success at the probe level was found to be the GC content. It has been shown that oligonucleotide probes with 42% GC (i.e. close to the optimal GC content of 44% in the NimbleGen probe design) had the highest hybridization intensity at 42°C as compared to 30% and 56% GC [48]. Probes with high GC content, however, were not sensitive to mismatches and outperformed probes with optimal GC content when there were several mismatches between the hybridizing DNA fragment and the probe [41, 48]. We conclude that this approach is rather conservative at the SNP level and identifies only genes with high divergence and is likely to miss single SNPs.

Secondly, we were able to reliably detect genetic variation above the SNP level, such as segmental duplications and deletions. Genes and regions showing high signal intensity (putatively present in many copies) had higher coverage in the *Littorina* genome sequencing and some of them had been previously annotated as transposon-like elements in *Littorina* [38, 54]. Genomic regions with signal intensity at the background level (putative deletions) were absent in the *Littorina* genome assembly. In addition, hybridization of genomic DNA fragments to the transcriptome-based array provided information on the position of exon-intron boundaries.

To conclude, using an oligoarray platform, it is possible to detect large deletions, segmental duplications and high divergence between sequences. Owing to the rapid development of Next Generation Sequencing techniques, future methods to study genome divergence between closely related species and populations are likely to employ low coverage genome re-sequencing and reduced representation sequencing approaches. Still our analyses show that long oligonucleotide genomic arrays can be a useful tool for genotyping different types of genetic variation simultaneously, especially if probe length is optimized for SNP detection. For example, a 50-nt tiling array has been designed for single SNP genotyping in *Caenorhabditis elegans* [44] and a tiling array with various probe lengths has been used to screen for novel deletions, chromosomal breakpoints and SNPs in the fungus *Trichoderma reesei* [45].

### Genome divergence and phylogenetic relationships in closely related North-Atlantic *Littorina*species

The overall genome divergence pattern of the four closely related North-Atlantic *Littorina* species included here corresponds well to the phylogenetic reconstructions based on only a few genes [26, 31]. *Littorina fabalis*, which diverged from *L. saxatilis* 2-4 Mya, showed lower hybridization success to the *L. saxatilis* array and was clearly separated by PCA. *Littorina arcana* and *L. compressa*, with divergence times from *L. saxatilis* estimated to be 0.06-1.42 Ma, did not show separation from *L. saxatilis* by PCA when all genes were taken into account. However, there were a number of genes with significant signal variation among the three sibling species, and species separation was much more pronounced (both in number of significant genes and in PCA clustering) than between British and Swedish populations of *L. saxatilis*. This suggests that some genes have diverged but a large part of the genome still shares ancestral variation in these sibling species. This finding is in agreement with earlier reports on shared genetic variation in allozymes, nuclear introns and mtDNA [28, 29, 77–80].

Our genome-wide analysis could not resolve the trichotomy between these three species. On one hand, the overall hybridization success was lower in *L. compressa* than in the other two species, likely due to a higher sequence divergence. This would imply that *L. compressa* was first to split out within this group, as was suggested by Knight & Ward [77] and Wilding *et al.* [79, 80]. On the other hand, there were more genes with significant divergence between *L. arcana* and *L. saxatilis* than between *L. compressa* and *L. saxatilis.* Finally, our hierarchical clustering analyses based on all genes could not resolve the order of splits between these species, and the degree of separation by PCA was roughly similar for all pairwise species comparisons. Solving the phylogenetic relationships between these sibling species is further complicated by the fact that the number of diverged genes can reflect both the on-going process of lineage sorting [2] or divergent selection in newly formed species [12, 14]. The geographic ranges of *L. compressa* and *L. arcana* are much more limited than that of *L. saxatilis* and, when all three species co-exist on the same shore, their micro-zonal distributions are only partly overlapping [25]. This may indicate differences in ecological and microhabitat preferences between these species. Under diversifying selection some genes may have achieved higher divergence between *L. arcana* and *L. saxatilis*, while others have diverged more between *L. compressa* and *L. saxatilis*, which is supported by our comparison of the genes with pair-wise divergence between the species (see below). At this point we agree with Reid *et al.* [26, 31] that the phylogenetic relationships between these three species are best represented as a trichotomy, reflecting the fact that the two divergence events occurred recently and very close in time, and that their order cannot be resolved. Hence, we predict that if more genes are analyzed in the future, the genealogies will continue to produce conflicting phylogenies for this group.

Altogether, the neighbour-joining tree of the studied *Littorina* lineage based on the aCGH data is very similar to earlier phylogenetic trees based on a few loci for species relationships [26, 31] or even on single mtDNA locus for regional variation in *L. saxatilis* [28, 29]. Even discrepancies between markers, as in case of the three sibling species that had earlier led to conclusion of an unresolved trichotomy, have support by the observed genome-wide pattern. Thus, this study provides one of the first comparisons of genome-wide variation to single locus estimates. Together with other studies demonstrating the utility of, for example, mtDNA markers in phylogeny and phylogeography [81, 82], our results suggest that single-gene phylogenies can indeed be informative and reliable, and even in the future may serve as useful tools for at least pilot phylogenetic reconstructions.

### Geographic variation in *L. saxatilis*

The Spanish population of *L. saxatilis* appears to be genetically distinct from the two more northern populations almost to the same degree as the three sibling species included in this study (by PCA plots and the number of genes identified as diverged). This confirms the conclusion from mtDNA analyses of a long independent evolutionary history of the Spanish *L. saxatilis* population and divergence time estimates of approx. 0.25 Ma [28, 29]. Interestingly, a breeding experiment showed that crosses between Spanish and Swedish snails produce viable and fertile offspring (K. Johannesson, unpublished observation). The two other populations of *L. saxatilis*, from Britain and Sweden, did not show any divergence at all in our analyses. This agrees with a hypothesis that these populations were established through relatively recent, post-glacial colonization events from a shared refugium or refugia other than the Spanish coast [29].

Local forms of Crab and Wave ecotypes of *L. saxatilis* exist in Spain, Britain and Sweden and a key issue is whether or not these ecotypes have evolved repeatedly or have one common origin [33, 34]. Genome divergence of the Spanish populations from the two northern populations, detected in this study, clearly supports a recent finding [35] that the Crab and Wave ecotypes of Spain *vs.* Sweden/Britain, have evolved independently of each other and possibly from different genetic backgrounds, despite similar phenotypic characteristics.

### Rapidly evolving and duplicated genes in the studied *Littorina*lineage

Most of the genes showing significant divergence in the studied *Littorina* lineage were specific to pairs of species. This might be due to random accumulation of differences with time or due to species-specific selection regimes (and in which case we can identify genes and genome regions involved in adaptations of the different species). Moreover, 35 genes showed elevated divergence in all pair-wise species comparisons. These come from the transcriptome library and did not show similarity to any know proteins, probably due to their short length, and will be a focus in future studies.

We did not find any genes with significant array hybridization differences between Crab and Wave ecotypes. However, there is evidence for a genetic basis of ecotype differences [83–86] and of limited gene exchange between the ecotypes [37, 58, 59, 87, 88]. Given that our aCGH approach was not sensitive enough to detect single SNPs, our results suggest that the genetic variation behind the ecotype differences is likely to be at the level of single mutations in coding or regulatory sequences, that may have large phenotypic effects [89, 90]. Indeed, an earlier study detected SNP variation in transcript sequences between the ecotypes in Britain [36]. Another type of genetic variation that may facilitate adaptive divergence is chromosomal inversions. This has been suggested *e.g*. for ecotypes of *L. fabalis*, although direct evidence for it is lacking [91, 92]. Our aCGH method cannot provide any information on chromosomal inversions, and the importance of this mechanism in the evolution of *Littorina* ecotypes is yet to be investigated.

The hybridization pattern along the CH317-123M16 BAC-clone indicated an insertion-deletion polymorphism for a large genomic region in *L. saxatilis*. The deleted region, identified in this study, does not appear to contain any open reading frames [38]. The CH317-123M16 fragment has been identified previously by an AFLP-scan for outliers between the British ecotypes of *L. saxatilis* [37] and contains insertions of repeated transposable elements outside the putatively deleted regions [38]. Although our data support an insertion-deletion polymorphism and transposable elements in this region, we did not observe any differences between the ecotypes, and deletion variants appear to be common in both British and Swedish populations. An alternative explanation to the lack of hybridization to this fragment is that there is an artifact in BAC assembly.

In contrast to the cohort of rapidly evolving genes, multiple-copy genes were mostly shared between the studied *Littorina* species. We found evidence that the snail genome probably contains a high level of segmental duplications, as 23% of BAC regions had high signals, which is not surprising given the relatively large genome size of 1.3 Gbp [75]. Further, our data indicate that at least some of the duplicated regions in the *Littorina* genome are associated with transposable elements and repeats. High abundance of repeats and multiple-copy regions has been found in the recently published genomes of the mollusks *Conus bullatus* [93] and *Crassostrea gigas* [94]. Our analyses produced a list of over 2,000 genes that are likely to be present in multiple copies in the snail genome. However, due to the short length of transcripts used for the array design, several transcripts may correspond to the same gene [54], or different members of gene families may be represented by the same partial transcript. Thus, the number of duplicated genes in the snail genome will require further investigation.

A few percent of the analyzed genes appear to be duplicated in only one species, suggesting that there are CNVs between these closely related littorinid species. Finally, in the comparisons of duplicated genes we found some indication for CNVs between the Crab and Wave ecotypes. This will require further confirmation since these differences were not significant in the ANOVA, but they are generally in agreement with the earlier observation of transposable element variation associated with the British ecotypes [38].

Presently, the low annotation success of *Littorina* transcripts (below 10%, see [54] for possible reasons) limits the biological and functional information that we can extract from the present dataset, i.e. we do not know the function of genes that show signs of duplication and/or rapid divergence in the analyzed littorinid species. However, on-going *de novo* genome sequencing and several transcriptome characterization projects in *Littorina* will potentially change the situation in the near future. When these resources become available, the next step will be to map the candidate sequences, identified in the present study, to annotated genes. Further, the identified candidate genes with high divergence between the species will be used in re-sequencing studies in order to distinguish signatures of diversifying selection from incomplete lineage sorting.

## Conclusions

In the present study we showed that aCGH can be performed for non-model organisms by hybridization of labeled genomic DNA to transcriptome oligonucleotide arrays. We used this approach to study genome divergence in the North-Atlantic intertidal species of *Littorina* snails (*L. fabalis*, *L. arcana*, *L. compressa* and *L. saxatilis*) as well as among geographic populations of *L. saxatilis*, representing radiation events from approximately 2-4 Mya to very recent post-glacial events. In addition, we looked for genome differentiation between the two common ecotypes of *L. saxatilis*.

1. By comparisons of probe hybridization signals to the *Littorina* genome draft and the direct re-sequencing of probes we showed that aCGH can successfully detect copy number variations, segmental deletions and high sequence divergence (i.e. at the level of several nucleotides per 60 nt probe length). However, the method is not sensitive enough to detect single SNPs.
2. Overall, genomic hybridization patterns are in agreement with the single-gene phylogenies and molecular estimates of divergence times for these closely related species, which lends credibility to the numerous phylogenies that have been, and still are, based on only one or a few genes.
3. We were not able to resolve conflicting phylogenies produced by different markers for the three sibling species *L. saxatilis*, *L. arcana* and *L. compressa*. We hypothesize that there is high variation between individual gene genealogies in this group owing to very incomplete processes of lineage sorting and/or diversifying selection, and the order of the species splits may not be resolvable.
4. We detected a surprisingly high level of genomic divergence between the Spanish and the British/Swedish populations of *L. saxatilis*, in fact similar to divergences among the sibling species. This lends strong support to the hypothesis of long isolation of the Spanish populations and independent evolution of snail ecotypes in Spain and in the two other regions.
5. While there are multiple sources of evidence for a genetic basis of the *L. saxatilis* ecotype variation, this variation could not be detected by the present method and is likely to be on the level of single SNPs.
6. Finally, we found 35 genes that could be candidates for rapidly evolving genes within the entire *Littorina* (*Neritrema*) lineage. However, many more genes showed elevated divergence between pairs of the species compared. On the other hand, duplicated genes were mainly shared between all the species studied here. Our analyses indicated a high degree of segmental duplication in the *Littorina* genome (23% of the analyzed genomic fragments) and likely to be associated with transposable elements.

To conclude, the results of the present study provide new information on the sensitivity and potential use of long oligonucleotide arrays for genotyping in non-model organisms. Applying this method to *Littorina* sp. provided the first insight into genome evolution of a recently speciated genus and an ongoing radiation within one of the species, *L. saxatilis*.

## Availability of supporting data

The full dataset from the oligonucleotide aCGH experiment has been submitted to the NCBI gene Expression Omnibus [95, 96] under the accession ID GSE59825.

## Electronic supplementary material

Additional file 1: Figure S1: Example of signal distributions after ANOVA and RMA normalizations. (PDF 56 KB)

Additional file 2: Figure S2: Genome sequencing coverage for genes with normal and high aCGH signal intensities. (PDF 49 KB)

Additional file 3: Table S1: Lists of genes with high aCGH signal in *Littorina* species, populations and ecotypes. (XLSX 454 KB)

Additional file 4: Table S2: Lists of genes with significant (q = 0.05) aCGH differences between *Littorina* species. (XLSX 228 KB)

## Abbreviations

aCGH: Array comparative genomic hybridization
AFLP: Amplified fragment length polymorphism
ANOVA: Analysis of variance
BAC: Bacterial artificial chromosome
CNVs: Copy number variants
mtDNA: Mitochondrial DNA
PCA: Principal component analysis
SNPs: Single nucleotide polymorphisms.
