# Supplementary material for: Species and gene divergence in Littorina snails detected by array comparative genomic hybridization
Source: BMC Genomics. 2014 Aug 18;15(1):687. doi: 10.1186/1471-2164-15-687 (PMC4148934; doi:10.1186/1471-2164-15-687)
Supplement: Supplementary file 1 — Additional file 1: Figure S1: Example of signal distributions after ANOVA and RMA normalizations. (PDF 56 KB) [file 12864_2014_6379_MOESM1_ESM.pdf]

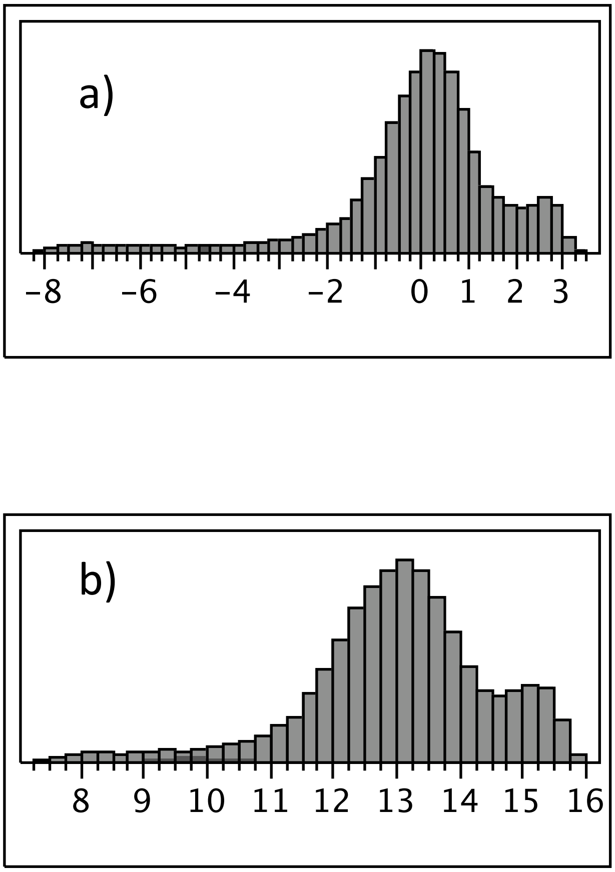

Fig. S1. Comparizon of ANOVA and RMA normalization methods for *Littorina* aCGH.

Overall signal distributions in one sample after normalization by a) ANOVA and b) RMA methods. X axis represents log2-transformed signals with subtracted average (a) or log2-transformed signals after RMA normalization (b). Note very similar shapes of the two distributions. Correlation between signal levels per gene model, produced by the two normalization methods, is R^2=0.967, p<0.00001.
