# Supplementary material for: Species and gene divergence in Littorina snails detected by array comparative genomic hybridization
Source: BMC Genomics. 2014 Aug 18;15(1):687. doi: 10.1186/1471-2164-15-687 (PMC4148934; doi:10.1186/1471-2164-15-687)
Supplement: Supplementary file 2 — Additional file 2: Figure S2: Genome sequencing coverage for genes with normal and high aCGH signal intensities. (PDF 49 KB) [file 12864_2014_6379_MOESM2_ESM.pdf]

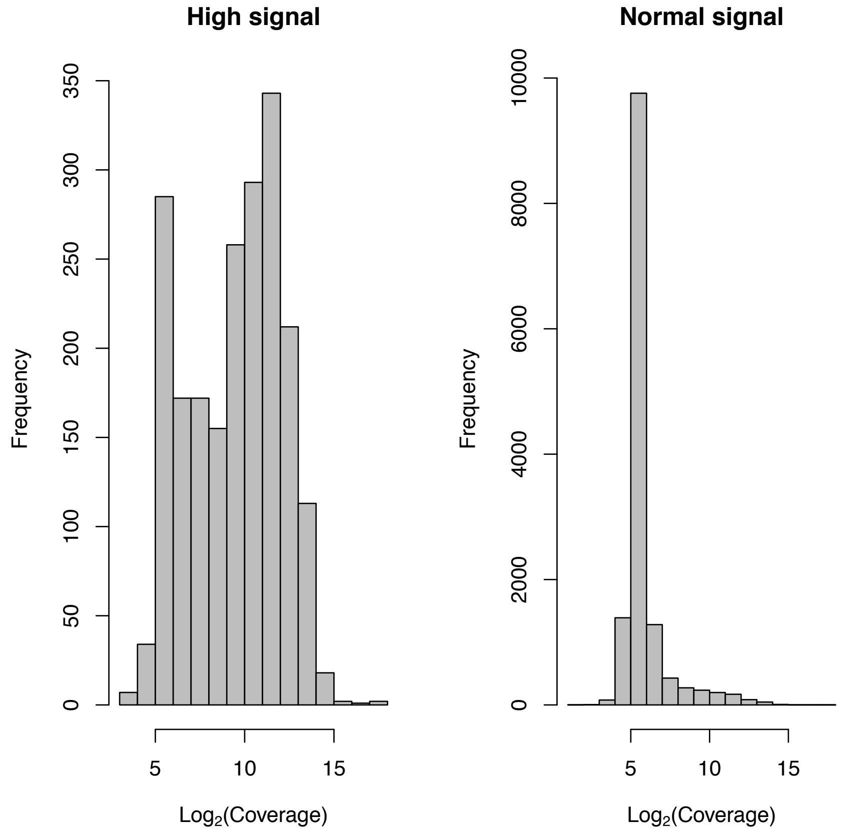

Fig. S2. Genome sequencing coverage for genes with normal and high aCGH signal intensities .

Coverage in the *Littorin*a genome sequencing was calculated for 2,067 genome contigs identified by aCGH as multiple-copy (”High signal”) and for 13,956 contig identified by aCGH as single-copy (”Normal signal”). Coverage is given at log2 scale, i.e. 5 corresponds to x32, 10 to x1024 and 15 to x32,768 times coverage. Median coverage for single-copy genes was x50 (5.64 at log2 scale). For multiple-copy genes it was x880 (9.8 at log2 scale, or 18 copies). Expected genome sequencing coverage was x67.
